# Distribution and Location of BEVs in Different Genotypes of Bananas Reveal the Coevolution of BSVs and Bananas

**DOI:** 10.3390/ijms242317064

**Published:** 2023-12-02

**Authors:** Xueqin Rao, Huazhou Chen, Yongsi Lu, Runpei Liu, Huaping Li

**Affiliations:** Guangdong Province Key Laboratory of Microbial Signals and Disease Control, College of Plant Protection, South China Agricultural University, Guangzhou 510642, China; raoxq@hotmail.com (X.R.); 13124947171@163.com (H.C.); sisi1187934@163.com (Y.L.); 15638610912@163.com (R.L.)

**Keywords:** *Musa*, banana streak virus (BSV), banana endogenous badnavirus sequences (BEVs), coevolution

## Abstract

Members of the family *Caulimoviridae* contain abundant endogenous pararetroviral sequences (EPRVs) integrated into the host genome. Banana streak virus (BSV), a member of the genus *Badnavirus* in this family, has two distinct badnaviral integrated sequences, endogenous BSV (eBSV) and banana endogenous badnavirus sequences (BEVs). BEVs are distributed widely across the genomes of different genotypes of bananas. To clarify the distribution and location of BEVs in different genotypes of bananas and their coevolutionary relationship with bananas and BSVs, BEVs and BSVs were identified in 102 collected banana samples, and a total of 327 BEVs were obtained and categorized into 26 BEVs species with different detection rates. However, the majority of BEVs were found in Clade II, and a few were clustered in Clade I. Additionally, BEVs and BSVs shared five common conserved motifs. However, BEVs had two unique amino acids, methionine and lysine, which differed from BSVs. BEVs were distributed unequally on most of chromosomes and formed hotspots. Interestingly, a colinear relationship of BEVs was found between AA and BB, as well as AA and SS genotypes of bananas. Notably, the chromosome integration time of different BEVs varied. Based on our findings, we propose that the coevolution of bananas and BSVs is driven by BSV Driving Force (BDF), a complex interaction between BSVs, eBSVs, and BEVs. This study provides the first clarification of the relationship between BEVs and the coevolution of BSVs and bananas in China.

## 1. Introduction

*Banana streak virus* belongs to the genus *Badnavirus* in the family of *Caulimoviridae*. Banana streak virus (BSV) is a double-stranded-DNA (dsDNA) virus with a rod-shaped structure and a monopartite circular genome of approximately 6.8–9.2 kbp, which comprises three open-reading frames (ORFs) [1]. The viral ORF I and II encode two small proteins [2], while ORF III encodes a large polyprotein that includes movement protein (MP), coat protein (CP), aspartic protease (AP), reverse transcriptase (RT), and ribonuclease H (RNase H). The RT/RNase H gene is the most conserved region [3].

The *Caulimoviridae* family is known for integrating the most abundant endogenous pararetroviral sequences (EPRVs) into the genomes of monocotyledonous and dicotyledonous plants [4,5]. Currently, *Musa* genomes contain two integrated BSV sequences, namely endogenous BSV (eBSV) and banana endogenous badnavirus sequences (BEVs). eBSVs contain active and inactive BSV sequences [5]. In certain situations, active eBSVs have the potential to generate infectious BSVs, resulting in systemic infection in bananas [6,7,8,9], while inactive eBSVs cannot give rise to infective BSVs due to their genetic mutations or replication defects [5,8]. eBSVs exist only in the genome of *M. balbisiana* (B genome) [1,8,10]. However, BEVs are deemed as viral fossils [5,11], which are unable to produce BSVs due to their limited genetic capacity [10,12]. The integration of BEVs into the host genome occurs through illegitimate recombination during the repair of host DNA [13,14]. These viral gene sequences for integration are permanently inserted into the host genome via pseudogenization [12,15,16] and thereafter maintained by natural selection [5,16].

Currently, the demarcation criteria for a new badnavirus species is an 80% divergence threshold in the nucleotide sequence of the RT/RNase H region, recommended by the International Committee on Taxonomy of Viruses (ICTV) [17]. As the recommendations, BSVs are grouped into three clades [12,18,19,20]: Clade I comprises various BSVs infecting bananas around the world, while Clade II solely includes BEVs. Most of the BSVs in Clade III were found in Uganda [12,16]. However, Harper et al. [18] identified 15 BSVs in bananas from Uganda, five of which (BSUCV, BSUDV, BSUFV, BSUGV, and BSUHV) were confirmed as BEVs and renamed BEV UC, UD, UF, UG, and UH [20]. Additionally, Geering et al. [10] identified a total of 33 BEVs, some of them categorized in Clade I. Presently, there are eight named BEVs, including BEV NGA [21], UC, UD, UF, UG, UH, P, and Q [20].

Banana cultivars are mainly derived from the intraspecific and interspecific hybridization and polyploidization of *M. acuminata* (A genome) and *M. balbisiana* (B genome), generating various genotypes of bananas [21,22]. BEVs were presented in the genomes of *M. acuminate* (A genome), *M. balbisiana* (B genome), and *M. schizocarpa* (S genome) [12,15,16,21,23]. Moreover, BEV UC, UF, and UG are distributed widely across the genomes of diploid and polyploid cultivars of *M. acuminata* and *M. balbisiana* [5,10,20]. D’Hont et al. [21] confirmed the presence of BEVs at 24 loci on 10 out of 11 chromosomes in *M. acuminate* DH Pahang. Interestingly, some BEVs were associated with specific banana genomes. For example, BEV UD, UH, and NGA were found in the genome of *M. acuminata*, while BEV UH was detected in the genome of *M. schizocarpa*. Furthermore, BEV P and Q were solely present in the genome of M. *balbisiana* [20].

EPRVs are distributed widely in plant genomes and recognized as a significant component of host genomes [7,24,25,26]. Next-generation sequencing technology has revealed an increase in the number of EPRVs within the *Caulimoviridae* family. To this date, no full BSVs have been associated with these BEVs [21,27]. The current research on BEVs has mainly addressed their identification, genetic diversity, and evolution [5,10,12,20]. D’Hont et al. [21] found some BEVs distributed on the *M. acuminate* DH Pahang chromosome while examining the evolution of the banana and monocotyledons. In addition, Chabannes et al. [20] analyzed the coevolution between badnaviral sequences and banana. 

It is well known that China is one of the origin place of the bananas, and diverse cultivars of bananas are grown in South China [28,29]. With the increasing number of BEVs identified, it is essential to objectively determine the evolutionary relationship between BEVs, BSVs, and bananas. However, the distribution of BEVs among the primary banana cultivars in China remains uncertain. To examine the distribution of BEVs in China’s primary cultivated bananas and the evolution of BSVs and bananas, a total of 327 BEVs were obtained by PCR and cloning from 102 banana samples collected from the different genotypes of bananas in China. The distribution and genetic diversity of BEVs in different genotypes of bananas were assessed through bioinformatics software, followed by an analysis of the coevolutionary relationships between BEVs, BSVs, and bananas. According to the findings of this study and previous research, we proposed that the BSV Drving Force (BDF), composed of BSVs, eBSVs, and BEVs, was the force for the coevolution of BSVs and bananas. This study provides a scientific foundation for further investigating the coevolution of BSVs and bananas. Based on the classification criteria of *Badnavirus*, BEVs had been classified as tentative genera [10,20]. In this study, BEV species represented tentative species.

## 2. Results

### 2.1. Detection of BEVs

**Figure 1:**
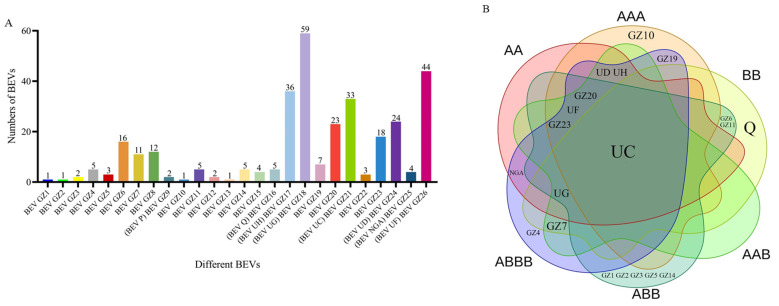
Detection of BEVs from different genotypes of bananas. (**A**) Different BEVs. (**B**) Six-way Venn diagram of BEVs among different A and B genotypes of bananas.

In this study, a total of 355 different sequences were obtained by PCR amplification, cloning, and sequencing from 102 samples of bananas. According to the ICTV classification standard of badnaviruses, 28 BSVs and 327 BEVs were found. A total of 28 BSVs belonged to 5 species of BSV, namely banana streak OL virus (BSOLV), banana streak GF virus (BSGFV), banana streak MY virus (BSMYV), banana streak IM virus (BSIMV) and banana streak VN virus (BSVNV). These 327 BEVs were categorized into 26 species, tentatively designated as BEV GZ1–GZ26 (Figure 1), including eight known BEVs such as BEV GZ9 (BEV P), BEV GZ16 (BEV Q), BEV GZ17 (BEV UH), BEV GZ18 (BEV UG), BEV GZ21 (BEV UC), BEV GZ24 (BEV UD), BEV GZ25 (BEV NGA) and BEV GZ26 (BEV UF), 3 new BEVs (BEV GZ2, GZ5, and GZ13), and 15 unnamed BEVs in the GenBank. Specifically, it appears that BEV UG, UF, UH, and UC had relatively high detection rates of 18.04%, 13.46%, 11.01%, and 10.09% (Figure 1A), respectively, compared to the other BEVs. This suggested that these BEVs were more commonly found in bananas. However, the remaining BEVs had lower frequencies, indicating that they were less prevalent in bananas. The distribution of BEVs in bananas seems to be uneven.

### 2.2. BEVs in Different Genotypes of Bananas

The analysis of BEVs revealed that various species and numbers of BEVs were present in different genotypes of bananas (Figure 1B). Eight BEVs, namely BEV GZ20, GZ23, NGA, UC, UD, UF, UH, and UG, were found in AA bananas. Meanwhile, eight BEVs were demonstrated in AAA bananas, specifically BEV GZ10, GZ19, GZ20, GZ23, UC, UD, UF, and UH. Six BEVs were displayed in BB bananas, such as BEV GZ6, GZ7, GZ11, Q, UC, and UG. A total of eight BEVs were identified in AAB bananas, namely BEV GZ7, GZ20, GZ23, UC, UF, UD, UG, and UH. In ABB bananas, a total of twelve BEVs were identified, specifically BEV GZ1, GZ2, GZ3, GZ5, GZ6, GZ11, GZ14, GZ20, GZ23, UC, UF, and UG. Furthermore, eleven BEVs were found in ABBB bananas, such as BEV GZ4, GZ7, GZ19, GZ20, GZ 23, NGA, UC, UD, UF, UG, and UH.

The common and distinct BEVs were identified in AA and AAA bananas. The shared BEVs in both AA and AAA bananas were BEV UD, UH, GZ20, UF, GZ23, and UC. The exclusive BEVs in AA bananas were BEV NGA and UG, while the unique BEVs in AAA bananas were BEV GZ10 and GZ19.

Compared to other A-genome bananas (AAA, AAB, ABB, ABBB), the same and different BEVs were showed in AA bananas. Four BEVs shared among AA, AAA, AAB, ABB, and ABBB bananas, such as BEV GZ20, GZ23, UC, and UF. Four distinct BEVs (BEV NGA, UD, UH, and UG) were found in AA bananas. The different BEVs, such as BEV GZ10, GZ19, UD, and UH were identified in AAA bananas. The specific BEVs of AAB bananas were BEV GZ7, UD, UG, and UH. Eight unique BEVs (BEV GZ1, GZ2, GZ3, GZ5, GZ6, GZ11, GZ14, and UG) were found in ABB bananas. Meanwhile, the distinct BEVs of ABBB bananas were BEV GZ4, GZ7, GZ19, NGA, UD, UG, and UH.

The B-genome bananas (AAB, ABB, ABBB) shared the BEVs with BB bananas, including BEV UC and UG. The unique BEVs of BB bananas comprised BEV GZ6, GZ7, GZ11, and Q. The specific BEVs such as BEV GZ7, GZ20, GZ23, UF, UD, and UH were found in AAB bananas. The unique BEVs of ABB bananas were BEV GZ1, GZ2, GZ3, GZ5, GZ6, GZ11, GZ14, GZ20, GZ23, and UF. The specific BEVs of ABBB bananas were GZ4, GZ7, GZ19, GZ20, GZ23, NGA, UD, UF, and UH.

Some BEVs were shared in AA and BB bananas but they also had different BEVs. BEV UC and UG were found in both AA and BB bananas. However, specific BEVs, including BEV GZ20, GZ23, NGA, UD, UF, and UH, were shown in AA bananas, while BEV GZ6, GZ7, GZ11, and Q, as the specific BEVs, were identified in BB bananas.

The identical and different BEVs were found among A and B heterozygous bananas (AAB, ABB, and ABBB) as well as AA and BB bananas, respectively. BEV GZ20, GZ23, UF, UC, and UG were identified in A and B heterozygous bananas (AAB, ABB, and ABBB) and AA bananas. Compared to A and B heterozygous bananas (AAB, ABB, and ABBB), specific BEVs were present in AA bananas, namely BEV NGA, UD, and UH. On the other hand, BEV UC and UG were shared among A and B heterozygous bananas (AAB, ABB, and ABBB) and BB bananas, whereas BEV GZ6, GZ7, GZ11, and Q, were found in BB bananas, but not in A and B heterozygous bananas (AAB, ABB, and ABBB).

In particular, only BEV UC was shared among AA, AAA, BB, AAB, ABB, and ABBB bananas. No particular BEVs were present in AA or AAB bananas. The unique BEV GZ10 was shown in AAA bananas, while BEV Q was only found in BB bananas. Multiple unique BEVs, including BEV GZ1, GZ2, GZ3, GZ5, and GZ14, were shown in ABB bananas, whereas BEV GZ4 was found in ABBB bananas (Figure 1B). These results indicated that there were distinct BEVs in bananas with various genotypes. However, A and B heterozygous bananas had more BEVs in common with AA bananas than those with BB bananas.

### 2.3. Southern Blot Analyses of BEVs

**Figure 2:**
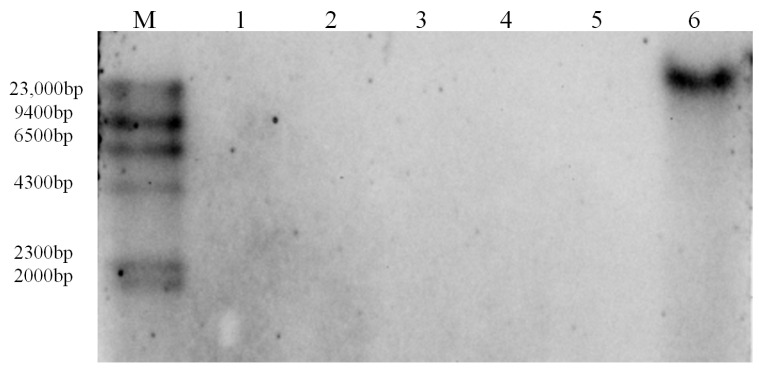
Southern blot analyses of BEV GZ2. (M) Digoxigenin-Labeled Marker; (1) Brazilian (AAA); (2) Williams (AAA); (3) Fenza 1(ABBB); (4) Guangfen 1 (ABB); (5) Jinfen (ABB); (6) Dajiao (ABB).

To determine the endogeny of BEVs, BEV GZ2 with low detection rate were analyzed by Southern blot. The results indicated that the hybrid signals of BEV GZ2 were present in Dajiao (ABB) with the same size as the total DNA of the banana genome (Figure 2). However, no hybrid signal was detected in the other five banana cultivars (Brazilian, Williams, Fenza 1, Guangfen 1, Jinfen), suggesting that BEV GZ2 are integrated into the genome of Dajiao (ABB).

### 2.4. Sequence Analyses between BEVs and BSVs

**Figure 3:**
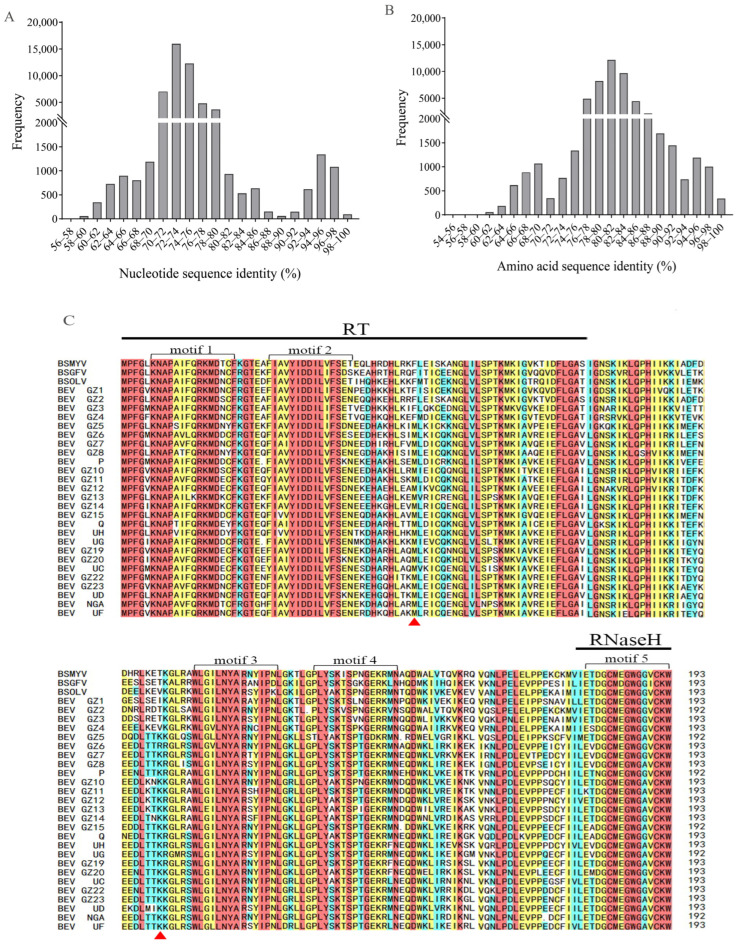
Alignment of nucleotide sequences and amino acid sequences of BEVs based on the partial RT/RNase H gene. (**A**) Frequency distribution of pairwise nucleotide sequence identities of the BEVs. (**B**) Frequency distribution of pairwise amino acid sequence identities of the BEVs. (**C**) Alignment of amino acid sequences of BEVs and BSVs. Red indicates that the sequence identity was 100%, yellow represents that the sequence identity was greater than 75%, blue shows that the sequence identity was greater than 50%, white expresses that the sequence identity was less than 25%, and a red triangle indicates the conserved sites of BEVs.

To assess the sequence variation among different BEVs species, we analyzed the nucleotide sequences and amino acid sequences of BEVs in the present study. Our results (Figure 3A) revealed that BEVs exhibited a range of nucleotide sequence identities from 57.4% to 100.0%. Notably, there were two distinct peaks of nucleotide sequence identity, 72–76% and 94–98%, which reflected relationships between and within BEVs species. Additionally, amino acid sequence analyses revealed that BEVs showed 54.9–100.0% identity. The identities of amino acid sequences between BEVs species were mainly 78.0–84.0%, while those within BEVs species were mainly 94.0–98.0% (Figure 3B). In addition, both BEVs and BSVs possessed five conserved motifs. Motif 1 and motif 2 were situated in the RT region [30], whereas motif 3 and motif 4 were, respectively, located in the intergenic region of RT and RNase H, and motif 5 was present at the 5′ terminus of the RNase H region. When comparing to BSVs, it was noteworthy that BEVs had two conserved amino acids that differed from BSVs. One was methionine, located at the 3′ end of RT, the other was lysine, located in the intergenic region of RT and RNase H (Figure 3C). The results indicated that both BEVs and BSVs shared five common motifs. This was the first time that BEVs were found to have two unique amino acids that distinguish them from BSVs.

### 2.5. Phylogenetic Analyses of BEVs

**Figure 4:**
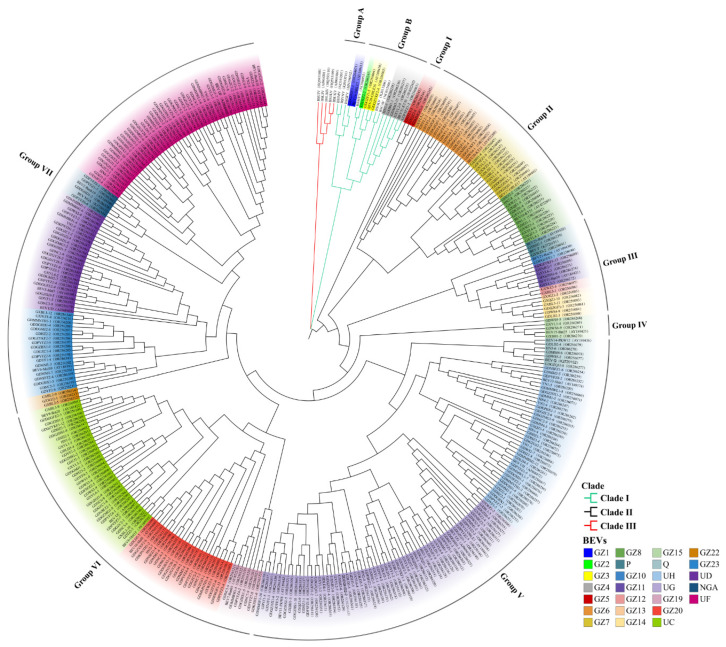
Phylogenetic tree constructed based on the partial sequences of RT and RNase H genes.

To investigate the phylogenetic relationships among the BEVs obtained in this study and the BSVs and BEVs from GenBank, we constructed a phylogenetic tree based on the partial sequences of RT and RNase H genes (Figure 4). The results showed that all of the BEVs in this study and the BSVs and BEVs obtained from GenBank could be grouped into three clades: Clade I, II, and III. Clade I contained both BEVs and BSVs, Clade II only included BEVs, while Clade III consisted of BSVs from East Africa [10,12].

Nucleotide sequence homology of BEVs and BSVs within the clades was analyzed. Clade I was divided into two subgroups, namely Group A and Group B. Group A included BEV GZ1, BEV28 (OBLE7), and BSIMV, BSVNV, and BSGFV, which clustered together with nucleotide sequence identities ranging from 68.7% to 99.8%. Notably, BEV GZ1 shared 99.8% sequence identity at the nucleotide level with BEV28 (OBLE7). However, BEVGZ2, GZ3, GZ4, BEV29 (OBLE36), BEV33 (KT30), BSOLV, BSMYY, and BSCAV belonged to Group B, with nucleotide sequence identities ranging from 63.4% to 98.1%. In particular, the nucleotide sequences of BEV GZ3 and BEV29 (OBLE36) had 98.1–99.2% identity, while those of BEV GZ4 and BEV33 showed 88.2–93.5% identity.

Clade II, which consisted entirely of BEVs, was the largest clade and was further divided into seven groups: Group I, II, III, IV, V, VI, and VII. The nucleotide sequence identities among BEVs within groups were analyzed, revealing a close phylogenetic relationship.

Group I consisted of different BEV GZ5 isolates, with the nucleotide sequence identity ranging from 91.8% to 98.2%.

Group II included BEVGZ6, GZ7, GZ8 and BEV17 (OBLE17), BEV18 (KT51), and BEV20 (KT23), with nucleotide identities between 72.5% and 99.2%. BEV GZ6 and BEV20 (KT23), BEV GZ7 and BEV17 (OBLE17), BEV GZ8 and BEV18 (KT51) clustered in the same branch with nucleotide sequence identities of 84.1–99.2%, 82.7–99.4%, and 89.5–99.2%, respectively.

Group III was composed of several BEVs, including BEV P, GZ10, GZ11, GZ12, GZ13, GZ14, BEV21 (Bat19), BEV23 (Bat36), and BEV25 (Bat10), which had 71.5–98.8% sequence identity at the nucleotide level. Notably, BEV P and BEV25 (Bat10), BEV GZ10 and BEV23 (Bat36), and BEV GZ11 and BEV21 (Bat19) clustered together with 94.1–98.8%, 92.9%, and 85.8–99.6% nucleotide identities, respectively.

Group IV consisted of BEV GZ15 and BEV15 (Bat 25), with nucleotide identities ranging from 94.8% to 99.7%. 

Group V included BEV Q, UH, UG, and BEV11 (PKW8), BEV12 (OBLE3), BEV13 (Shiz2), and BEV14 (PKW12) with nucleotide identities ranging from 72.7% to 99.7%. BEV Q and BEV14 (PKW12), as well as BEV UH and BEV13 (Shiz 2), were clustered in the same branch with nucleotide identities of 89.6–99.7% and 93.0–98.1%, respectively, whereas BEV11-PKW8 and BEV12 (OBLE3) were grouped in two different branches of BEV UG with nucleotide identities of 86.0–95.7% and 94.1–98.5%, respectively.

Group VI consisted of BEV GZ19, GZ20, UC, BEV7 (Bank9), BEV8 (Mal15), BEV9 (Batu20), and BEV10 (Mal8), displaying nucleotide identities ranging from 74.4% to 98.9%. BEV GZ19 and BEV7 (Bank9), BEV GZ20 and BEV10 (Mal8) shared the same branch with nucleotide identities of 97.2–98.5% and 95.7–98.3%, respectively. BEV8 (Mal15) and BEV9 (Batu20) belonged to different branches of BEV UC, with nucleotide identities of 84.0–97.3% and 93.3–99.0%, respectively.

Group VII contained BEV GZ22, GZ23, UD, NGA, UF, and BEV1 (Bank10), BEV2 (Bank19), BEV5 (Mal3), and BEV6 (Mal10). They shared nucleotide identities ranging from 75.4% to 99.1%. Among them, BEV GZ23 and BEV6 (Mal10), BEV UD and BEV5 (Mal3), BEV NGA and BEV2 (Bank19), and BEV UF and BEV1 (Bank10) were located in the same branch with the nucleotide identities of 86.9–98.3%, 91.3–99.0%, 95.7–97.2% and 80.6–99.0%, respectively.

### 2.6. Relationship between BEVs in Different Clades and the Genotypes of Bananas

Clade I consisted of Group A and Group B. Both of them had BSVs and BEVs. However, Group A contained solely BEV GZ1, while Group B included BEV GZ2, GZ3, and GZ4. Importantly, BEV GZ1, GZ2, and GZ3 were found in ABB bananas, whereas BEV GZ4 was present in ABBB bananas.

In Clade II, BEVs were divided into three types. The first type was the common BEVs shared by six genotypes of bananas (AA, AAA, BB, AAB, ABB, and ABBB), such as BEV UC. The second one referred to BEVs that shared among several genotypes of bananas. For instance, BEV UG was common in bananas with genotypes AA, BB, AAB, ABB, and ABBB but not in AAA bananas. BEV UH and UF were common BEVs in AA, AAA, AAB, ABB, and ABBB bananas but not in BB bananas. The third type included BEVs that were specific to a certain genotype of banana. For example, BEV GZ10 was found in AAA bananas. In addition, Group V in Clade II contained a significant number of BEVs, while Groups V, VI, and VII showed a higher abundance of BEV UH, UG, UC, and UF, respectively. All the AA, AAB, and ABBB bananas contained BEV UH, UG, UC, and UF, while AAA bananas contained BEV UH, UC, and UF. ABB bananas were found to have BEV UC, UF, and UG, and BB bananas had BEV UC and UG. According to the phylogenetic analyses, BEVs in AAA, AAB, and BB bananas were found in Clade II, while BEVs in ABB and ABBB bananas were located in Clade I and Clade II.

### 2.7. Distribution and Locations of BEVs on Chromosomes of Different Genotypes of Bananas

As BEVs were closely related to the genotypes of bananas, the genome sequences of *M. acuminata* subsp. DH-Pahang (AA), *M. balbisiana* subsp. PKW (BB), and *M. schizocarpa* subsp. HN8 (SS) were downloaded from websites to determine the distribution and location of the BEVs discovered in this study.

#### 2.7.1. Distribution and Location of BEVs on the Chromosomes of *M. acuminata* subsp. DH-Pahang (AA)

**Figure 5:**
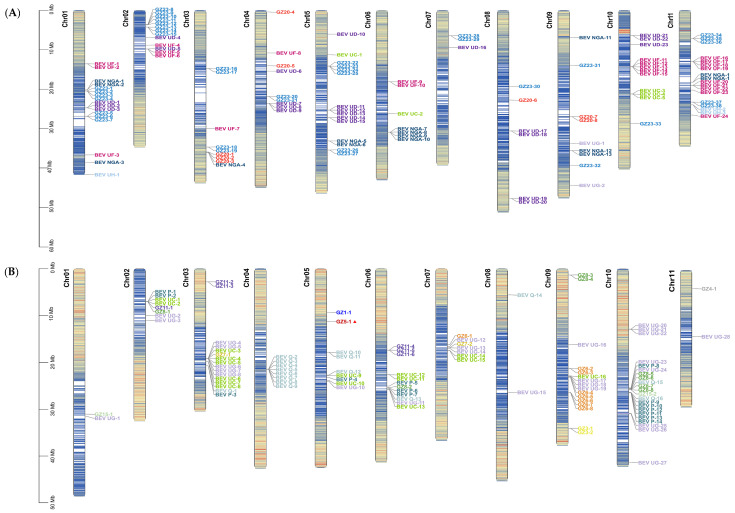

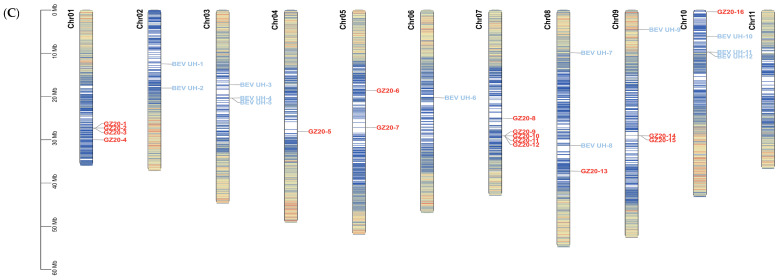
Distributions of BEVs on the chromosomes of bananas with different genotypes. (**A**) *M. acuminate* subsp. DH-Pahang (AA). (**B**) *M. balbisiana* subsp. PKW (BB). (**C**) *M. schizocarpa* subsp. HN8 (SS). The chromosome diagram shows the distribution of gene density. The color gradient from blue to red indicates a gradual increase in the quantity of genes.

BEVs in this study were analyzed for distribution across chromosomes (Chr) of *M. acuminata* subsp. DH-Pahang (AA). The results showed that eight BEVs species were located on the chromosomes of *M. acuminata* subsp. DH-Pahang, namely BEV GZ20, GZ23, NGA, UC, UD, UF, UG, and UH. A total of 118 loci were identified on 11 chromosomes for these BEVs. Interestingly, Chr 11 contained 19 BEVs loci, and Chr 01 had five BEV species, while Chr 07 only had 3 loci and two species of BEVs (Figure 5A).

The number of BEVs loci on *M. acuminata* subsp. DH-Pahang chromosomes varied from most to least in the following order: BEV GZ23, UF, UD, NGA, GZ20, UC, UH, and UG. BEV GZ23 had the most loci with 38, spanning ten chromosomes except Chr06. BEV UF had 24 loci on Chr01, Chr02, Chr03, Chr04, Chr06, Chr10, and Chr11. BEV UD, on the other hand, had 23 loci that were distributed across Chr01, Chr02, Chr04, Chr05, Chr07, Chr08, and Chr10. The locations of the other BEVs were listed in Table 1.

#### 2.7.2. Distribution and Location of BEVs on the Chromosomes of *M. balbisiana* subsp. PKW (BB)

The locations of BEVs in the chromosomes of *M. balbisiana* subsp. PKW (BB) were identified and are displayed in Figure 5B. A total of 14 species of BEVs, including BEV GZ1, GZ3, GZ4, GZ5, GZ6, GZ7, GZ8, GZ17, GZ18, P, Q, UC, UF, and UG, were found across the chromosomes of PKW (Figure 5B). In total, there were 106 loci of BEVs on 11 chromosomes of PKW. Chr10 had the most abundant loci, while Chr03, Chr05, and Chr06 had the most BEVs species. It was surprising that only BEV GZQ was present on Chr04.

The locations of BEVs on the chromosomes of *M. balbisiana* subsp. PKW were listed in descending order: BEV UG, UC/Q, P, GZ6/GZ8, GZ11, GZ3/GZ7/GZ15, GZ/GZ4/GZ5/ UF. Of these, 28 loci of BEV UG were identified on ten chromosomes of *M. balbisiana* subsp. PKW, except for Chr04. Additionally, 16 loci of BEV UC were distributed unevenly across Chr02, Chr03, Chr05, Chr06, Chr07, and Chr09. Similarly, BEV Q was present in 16 loci on Chr03, Chr04, Chr05, Chr06, Chr08, and Chr10. The other BEVs are shown in Table 2.

#### 2.7.3. Distribution and Location of BEVs on the Chromosomes of *M. schizocarpa* subsp. HN8 (SS)

A total of 28 distribution loci were found in *M. schizocarpa* subsp. HN8. There were only two BEV species (BEV GZ20 and BEV UH) across ten chromosomes except Chr11 (Figure 5C). BEV GZ20 was found to be located on Chr01, Chr04, Chr05, Chr07, Chr08, Chr09, and Chr10 with 16 loci. Notably, Chr07 showed the most loci with 5 loci. Similarly, BEV UH displayed 12 loci distributed across Chr02, Chr03, Chr06, Chr08, Chr09, and Chr10.

#### 2.7.4. Analyses of BEVs in Different Clades on the Different Chromosomes

Different BEVs were located on the chromosomes of AA, BB, and SS genotypes of bananas. The BEVs in Clade I were only found on the chromosomes of *M. balbisiana* subsp. PKW (BB), whereas those in Clade II were present on the different chromosomes of AA, BB, and SS bananas. BEV GZ1, GZ3, and GZ4 in Clade I were distributed on Chr05, Chr09, and Chr11 of *M. balbisiana* subsp. PKW (BB), they had only one or two loci. The four predominant BEVs in Clade II, namely BEV UG, UF, UH, and UC, were mainly located on the chromosomes of AA, BB, and SS bananas. BEV UG, UF, UH, and UC were scattered across the chromosomes of *M. acuminate* subsp. DH-Pahang (AA), and BEV UF was present at 25 loci found on seven different chromosomes, while the other three BEVs had less than 5 loci. The four main BEVs displayed distinct distribution on chromosomes of *M. balbisiana* subsp. PKW (BB), BEV UG was observed at 28 loci located across ten chromosomes, BEV UC was present at 16 loci on six chromosomes; BEV UF had only one location on Chr01, while BEV UH was not found in BB bananas. However, only BEV UH exhibited 12 loci dispersed throughout six chromosomes in *M. schizocarpa* subsp. HN8 (SS). The chromosomal mapping indicated BEVs in Clade I were exclusively located on the chromosomes of BB bananas, while BEVs in Clade II were present on the chromosomes of AA, BB, and SS bananas. Hence, the chromosomes of BB bananas contained BEVs in both Clade I and II.

### 2.8. Synteny Analyses of BEVs on the Chromosomes of Different Bananas

**Figure 6:**
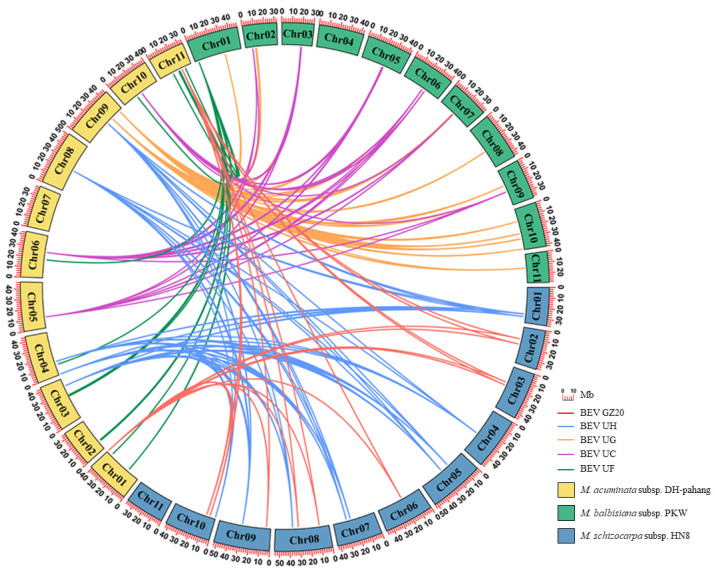
Collinear analyses of BEVs on the chromosomes of different bananas.

Collinear analyses were crucial for comprehending gene evolution. In this study, we investigated the syntenic relationships of BEVs among AA, BB, and SS bananas. The results indicated that five BEVs exhibited a syntenic relationship. For instance, three BEVs, including BEV UC, UF, and UG, showed a common presence in *M. acuminate* subsp. DH-Pahang (AA) and *M. balbisiana* subsp. PKW (BB), while two BEVs, namely BEV GZ20 and UH, were shared in *M. acuminate* subsp. DH-Pahang (AA) and *M. schizocarpa* subsp. HN8 (SS) (Figure 6). No common BEVs were observed between *M. balbisiana* subsp. PKW (BB) and *M. schizocarpa* subsp. HN8 (SS).

## 3. Discussion

### 3.1. BEVs in Different Genotypes of Bananas

EPRVs, as viral fossils, play a critical role in gene transfer from virus to host [11]. They have become an essential component of the genomes of host plants with time [31]. Recent studies have indicated that EPRVs are widely distributed throughout plant genomes [4,7,10,25]. In the current study, 327 BEVs were identified in different genotypes of the banana samples, indicating the significant integration of BEVs in diverse banana genomes. Chabannes et al. [20] discovered a close correlation between the distribution of eight BEVs and AA and BB bananas. Our findings were consistent with the results obtained by Chabannes et al. [20]. Interestingly, we observed fewer BEVs species in homozygous bananas (AA, AAA, and BB) than those in A and B heterozygous bananas (AAB, ABB, and ABBB). Moreover, heterozygous banana genomes with two or three B had more BEVs species than those with one B. During cross-breeding, the genomic structure and traits of heterozygous bananas were modified by gene introgression [10] or gene acquisition [32]. The introduction of new genes through cross-breeding promoted the diversification of gene functions and improved banana resistance to BSVs [33,34].

Some BEVs, such as BEV UG and UC, were more readily detectable in bananas. Our study supported the findings of previous studies [20,25,35]. The frequency of BEVs detected in different bananas was associated with their number of copies. Our findings confirmed that higher copies of BEVs generally resulted in easier detection. In addition, some bananas, such as ABB and ABBB, had distinct BEVs (Figure 1B), and these unique BEVs generally belonged to BEVs with a lower frequency of detection in the bananas [20]. This might be due to independent events of low-frequency integration [20], or a lack of significant amplification because of late integration [12]. Thus, low-copy BEVs had been found to have limited distribution and might only be present in certain genotypes of bananas. Additionally, the detection frequency of BEVs varied across the different bananas depending on the copy number of BEVs.

### 3.2. Genetic Diversity of BEVs

Badnaviruses exhibit significant genetic diversity and can be classified into three clades. Clade II solely comprises BEVs [12,20], with the exception of certain BEVs within Clade I [10]. In the current study, BEVs and BSVs were categorized into three clades. Notably, no BSVs were found in Clade III. BEVs were present in both Clade I and II, with most BEVs in Clade II, only a few in Clade I, which was consistent with the BEV classification of Geering et al. [10]. Some of the BEVs found in Clade I were identical to those found by Geering et al. [10], and three BEVs in Clade I were located in the Chr05, Chr09, and Chr11 of *M. balbisiana* subsp. PKW (BB). According to the results of Geering et al. [23] and Chabannes et al. [20], Clade I mainly included various BSVs that infected bananas, as well as eBSVs that had integrated into the banana B genome [16,36]. However, eBSOLV and eBSGFV were located on different regions of Chr01 of *M. balbisiana* subsp. PKW (BB genotype), respectively, whereas eBSIMV was located on Chr02 [8]. In the current study, the locations of BEVs in Clade I on the chromosomes of *M. balbisiana* subsp. PKW (BB) differed from those of eBSVs. Thus, the BEVs in Clade I could not be classified as either eBSVs or BSVs. However, the BEVs in Clade I showed a closer relationship to the BSVs in Clade I than the BEVs in Clade II, suggesting that the integration time of BEVs into the banana genome in Clade I was later than those in Clade II.

BEVs were mainly distributed in Clade II [20] and could be classified into distinct sub-clades [10]. The eight BEVs in Clade II, as reported by Chabannes et al. [20], were closely related to the BEVs described by Geering et al. [10], some of them had over 90% homology, while a few were located in different sub-clades. However, most of the BEVs obtained in the current study were classified into various groups within Clade II. The BEVs in the different groups were closely related, which supported previous research findings [12,15,20]. Although the nucleotide sequences varied significantly among different BEVs, the amino acid sequences were relatively conserved. In addition, we discovered that BEVs and BSVs shared three motifs with the amino acid sequences of RT and RNase H and that the RT and RNase H structural domains comprised the gag-pol core replication region of BSVs, which are essential components of all retrotranscription factors (including reverse transcriptase viruses and retrotransposons) [37]. The highly conserved RT and RNase H sequences appeared to strongly correlate with their enzymatic functions [38]. In the intergenic region of RT and RNase H, two common motifs were discovered that could potentially contribute to stabilizing the functions of the enzymes. Furthermore, we found that BEVs had two exclusive conserved amino acids distinct from those in BSVs. Further research is required to ascertain the roles of the two conserved amino acids.

### 3.3. Location of BEVs on Different Banana Chromosomes

With the increasing maturity of genomics and sequencing technology, the emergence of a large number of plant genomes has provided important data for studying the distribution and diversity of EPRVs. However, the number of integrated EPRVs varies among different host plants. Numerous EPRVs have been detected in citrus and tobacco [25,34]. However, the BEVs present in the banana were significantly fewer than the EPRVs found in the tobacco [34,39]. The integration patterns of BEVs on bananas might have differed from those of EPRVs on tobacco [12].

D’Hont et al. [21] identified 24 loci of BEVs on ten different chromosomes of *M. acuminata* subsp. Pahang (AA). Similarly, Gayral and Iskra-Caruana [12] discovered at least 27 integration events on AA, BB, and SS bananas. Chabannes et al. [20] revealed that the integration patterns of BEV UC and UG were more conserved in *M. balbisiana* (BB) than those in *M. acuminata* (AA). Our study showed that BEVs were present on most chromosomes of AA, BB, and SS bananas. In addition, BEVs exhibited greater diversity in *M. balbisiana* (BB) than those in *M. acuminata* (AA). There were more BEVs species on the chromosomes of *M. balbisiana* (BB) than on those of *M. acuminata* (AA). However, the BEVs distribution loci on the chromosomes of *M. balbisiana* (BB) were fewer than those on *M. acuminata* (AA), which could explain the lower polymorphism of BEVs observed in *M. balbisiana* (BB). Furthermore, we discovered fewer BEVs on the chromosomes of *M. schizocarpa* (SS) compared to both *M. acuminata* (AA) and *M. balbisiana* (BB). This finding was consistent with the results of Gayral and Iskra-Caruana [12], and the reason for this different distribution of BEVs might be related to the cultivation and variety selection of bananas. At the present, cultivated bananas are developed from wild bananas and selectively bred using conventional methods. These bananas primarily originated from A and B-genome bananas, with a smaller proportion derived from S-genome and T-genome bananas [40]. Most cultivated banana varieties had been developed through the intraspecific and interspecific hybridization of A and B bananas [21,29]. Therefore, BEVs showed more polymorphism in genetically heterozygous bananas with genotypes A and B than those in homozygous bananas with genotypes AA or BB. It was likely that BEVs were more prevalent in genetically heterozygous bananas with genotypes A and B.

We found that various BEVs were distributed unequally across banana chromosomes. For example, BEV UC and UG had more loci on the chromosomes of *M. balbisiana* subsp. PKW (BB) than those of *M. acuminata* subsp. Pahang (AA). In addition, most of the BEVs were distributed over Chr10 and Chr11 of both *M. balbisiana* subsp. PKW (BB) and *M. acuminata* subsp. Pahang (AA). BEV GZ23 and UG had the most loci, indicating that the distribution of BEVs was correlated not only with genotypes but also with chromosomes of bananas. Interestingly, multicopy BEVs appeared to form clusters on the banana chromosomes, showing that they might have integrated into the banana chromosomes before the divergence of the ancestral banana. This integration could have enabled the replication of BEVs through gene homologous recombination [41] or mediating chromosomal rearrangements [42]. As a result, multiple copies might have formed hotspots unevenly distributed across the chromosomes. In most plant genomes, EPRVs were often found near transposable elements [25]. Alternatively, they might reside in central regions that were rich in transposable elements but lack genes [43], or integrate into high-density gene regions that could have an impact on host gene structure and expression pathways [44], which could enable them to evade host plant elimination and facilitate coevolution between EPRVs and the host genome.

### 3.4. Coevolution of BEVs with BSVs and Bananas

**Figure 7:**
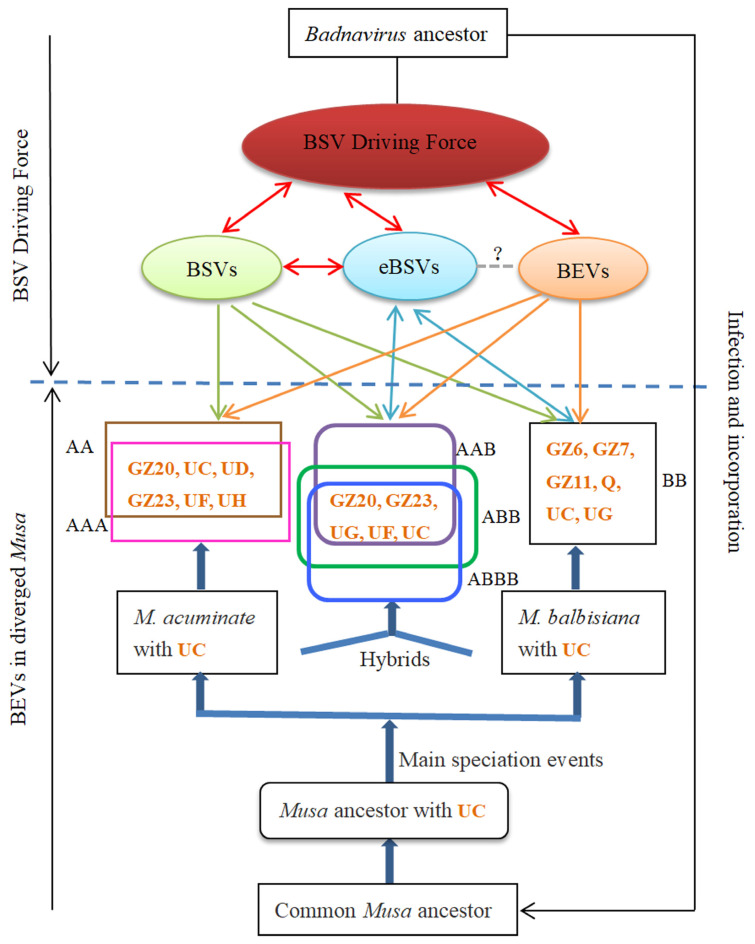
Coevolution dynamics hypothesis between bananas and BSVs. Red ellipse represents the BSV Driving Force (BDF). Red double arrows indicate that BSVs, eBSVs and BEVs interacted with each other. The BEVs in the box represent the BEVs integrated in different genotypes of bananas. The question mark (?) on the gray dotted line indicates an uncertain relationship between eBSVs and BEVs. Green arrows represent BSV-infected different genotypes of bananas. Blue double arrows indicate eBSVs in B-genome of bananas; some of them were able to be activated. Orange arrows show the distribution of BEVs in different banana chromosomes. The orange letters in the box represent BEVs integrated in different genotypes of bananas. The letters next to the boxes show different genotypes of bananas.

EPRVs in hosts record past viral infections [11] and have coevolved with their hosts, providing an opportunity to study the evolutionary correlation between viruses and hosts [14]. In the current study, the coevolution of bananas and BSVs in China was investigated (Figure 7) by analyzing the BEVs gene characteristics, BEVs distribution in different banana chromosomes, and the various cultivated bananas, together with the findings of previous research [9,20], to reveal the coevolutionary relationship between BSVs and bananas.

Both of BEVs and BSVs exhibited five common conserved motifs in the RT/RNase H and intergenic regions (Figure 3). The nucleotide sequence homology between eBSVs and corresponding BSVs exceeded 99%. Although the genomes of BSVs, eBSVs, and BEVs differed in size and function, they shared the RT/RNase H region [30,45] as well as common motifs, suggesting that they originated from a common badnavirus ancestor.

BSVs, eBSV, and BEVs could be transmitted from parent bananas to their offspring through tissue culture or cross-breeding. However, BSVs evolved faster than banana genomes [12]. BEVs were distributed throughout various chromosomes of different genotypes of bananas (Figure 5) and played a crucial role in the interaction and evolution of BSVs and bananas [12]. Although the sequences of the BEVs and eBSVs integrated into the banana genome were conservative on both sides, the structural variation was only observed in the viral sequences [15]. eBSVs, which acted as the reservoir for the BSVs [8], facilitated banana coevolution through the *M. balbisiana* genome [9]. In addition, BSVs, BEVs, and eBSVs facilitated the evolution of bananas while evolving themselves. Therefore, we propose that BDF, which is composed of BSVs, eBSVs, and BEVs, might serve as the driving force for the coevolution of BSVs and bananas.

BEV UC, as one of the common BEVs, was widely found in cultivated bananas with genotype AA, AAA, BB, AAB, ABB, and ABBB, as well as in *M. accuminate* subsp. Pahang (AA) and *M. balbisiana* subsp. PKW (BB). This suggests that BEV UC might originate from the *Musa* ancestor and is evidence of "molecular fossils" preserved on the chromosomes of the *Musa* ancestor after infection by the badnavirus ancestor, indicating that the integration time of BEV UC was earlier than the differentiation time of *M. acuminate* and *M. balbisiana*. This is consistent with the integration of BEV UC into the banana ancestor (*Eumusa* ancestor) proposed by Chabannes et al. [20].

BEVs GZ20, GZ23, UD, UF, and UH were common in AA and AAA bananas, while BEV GZ6, GZ7, GZ11, Q, and UG were found in BB bananas. BEV GZ20, GZ23, UF, and UG were shared among A and B heterozygous bananas (AAB, ABB, and ABBB), indicating that BEV GZ20, GZ23, and UF originated from AA bananas, while BEV UG was derived from BB bananas. The BEVs presented in the parents had been passed on to the heterozygous bananas (AAB, ABB, and ABBB) [10]. Although two syntenic relationships of BEVs (BEV UF and UG) existed in *M. acuminata* (AA) and *M. balbisiana* (BB) (Figure 5), the location analyses indicated a preference for the distribution of BEV UF and UG. AA bananas exhibited more BEV UF, while BB bananas had more BEV UG. It was consistent with the origin of BEV UF and UG in the A and B heterozygous bananas. This suggests that the integration time of BEVs GZ20, GZ23, UF, and UG into the chromosomes of *M. acuminata* and *M. balbisiana* occurred after the divergence of *M. acuminata* and *M. balbisiana*, and was later than the integration of BEV UC. In addition, some bananas had specific BEVs, such as BEV GZ10 presented in AAA bananas. These BEVs integrated into the banana chromosomes were later than those of BEVs GZ20, GZ23, UF, and UG, demonstrating that the integration time for different BEVs in the banana chromosomes varied.

However, the detection and location of some BEVs in different banana varieties with the same genotype did not show complete consistency; for instance, BEV UF was not detected in BB banana cultivars in this study, but was present in *M. balbisiana* subsp. PKW (BB). This divergence might be due to different integration events of the same BEVs in different banana cultivars with the same genotype. Therefore, there was incomplete consistency of BEVs among different banana cultivars with identical genotypes. BEVs in different banana cultivars demonstrated the diverse evolutionary processes of banana in China.

BSVs, BEVs, and eBSVs jointly drove the coevolution of bananas and BSVs. Infective BSVs derived from eBSVs [1,8,46] could be transmitted by mealybugs among different bananas [47], which not only accelerated the transmission of BSVs, but also contributed to the epidemic of banana streak disease [48,49]. Therefore, BDF represented a significant potential threat to the banana production in China, highlighting the emergence of banana streak disease and the urgent need for effective prevention and control measures of BSVs.

## 4. Materials and Methods

### 4.1. Plant Materials

During 2021–2022, a total of 102 banana samples were collected from the Guangdong, Guangxi, Yunnan, and Hainan provinces in China, including cultivated bananas with genotypes AA, AAA, BB, AAB, ABB, and ABBB, as well as some unknown genotypes. The details are presented in Table 3. The leaf samples were stored at −80 °C, while the banana suckers were planted in a greenhouse of the Plant virology laboratory, South China Agricultural University.

### 4.2. DNA Extractions of Banana Samples and Cloning and Sequencing

Total DNA was extracted from fresh or frozen banana leaves using the plant genome DNA extraction kit from Tiangen Co. (Beijing, China), following the recommended instructions, and then stored at −20 °C. The degenerate primer pairs Badv-RT-F/R (Badv-RT-F: 5′-ATGCCNTTYGGNNTNAARAAYGCNCC-3′; Badv-RT-R: 5′-CCAYTTRCANACNSCNCCCCANCC-3′) were designed based on the conserved sequences of the RT/RNase H region of badnavirus [50,51]. The extracted DNA was used as a template for PCR amplification via high-fidelity enzymes according to the manufacturer’s instructions (Vazyme, Nanjing, China). The amplified product had an estimated length of 579 bp. The PCR cycling conditions were 94 °C for 5 min followed by 35 cycles at 94 °C for 1 min, 52 °C for 1 min, 72 °C for 1 min, and a final extension of 72 °C for 10 min, and then stored at 4 °C. The PCR product was electrophoresed on a 1% agarose gel, and the target fragments were purified using the AP-GX DNA gel extraction kit (Axygen, Tewksbury, MA, USA) and cloned into pMD19T (Sanggon, Shanghai, China). Positive clones were identified using PCR. At least five positive clones per sample were chosen and sent to Sanggon (Shanghai, China) for sequencing. The classification of BEVs and BSVs was recommended by the ICTV [17,20]. The episomal BSVs were confirmed using improved immunocapture PCR [52] and banana genomic DNA was treated with RNase-free DNase I (Tiangen, Beijing, China) [20].

### 4.3. Southern Blot

To investigate the endogeny of BEVs, a Southern blot analysis was performed using the Roche DIG High Prime DNA Labeling and Detection Starter Kit II (Roche, Basel, Switzerland) following the manufacturer’s instructions. Genomic DNA from Brazilian (AAA), Williams (AAA), Fenza 1 (ABBB), Guangfen 1 (ABB), Jinfen (ABB), and Dajiao (ABB) varieties was extracted using the new plant genomic DNA extraction kit from Tiangen (Beijing, China). The probes of BEVs were synthesized using the Roche PCR DIG Probe Synthesis Kit (Roche, Basel, Switzerland). The total DNA of 40 μg was electrophoresed on 1% agarose gels and transferred overnight to the Hybond-N+ membrane (GE Healthcare, Little Chalfont, Buckinghamshire, UK). Nucleotides were then fixed to the membrane using a UV crosslinker (250 nm, 2 min), pre-hybridized for one hour at 42 °C, and hybridized overnight at the same temperature. Immuno-detection and imaging were performed according to the manufacturer’s instructions. A bio-macromolecular analyzer (Biowad-MPe, Bio-Rad, Hercules, CA, USA) was used for observation.

### 4.4. BEVs Sequence Analyses and Phylogenetic Analyses

The multiple sequences of BEVs obtained in this study were aligned using DNAMAN software (Version 7) to remove duplicates. The BEV sequences were analyzed using Clustal W in MEGA11 [53]; 327 BEVs in this study and 32 BEVs from GenBank were used to construct a phylogenetic tree using the neighbor-joining (NJ) method with 1000 bootstrap replicates. Additionally, the nucleotide and amino acid sequences of the BEVs were aligned using DNAMAN software (Version 7), respectively. Simultaneously, the conserved motifs of amino acid were analyzed through the MEME Suite (http://meme-suite.org/, accessed on 27 February 2023) and ProScan (http://www.ebi.ac.uk/interpro/search/sequence/, accessed on 27 February 2023), respectively.

### 4.5. Analyses of BEVs Distribution on Banana Chromosomes

The genomes of *M. acuminata* subsp. DH-Pahang (genotype AA, 443.29 MB) [54] and *M. schizocarpa* subsp. HN8 (genotype SS, 509.3 MB) [55] were obtained from the Banana Genome Center (https://banana-genome-hub.southgreen.fr/, accessed on 27 February 2023), and the genomes of *M. balbisiana* subsp. PKW (genotype BB, 464.64 MB) [56] were downloaded from GenBank, respectively. The banana genome database was constructed using the Blast Zone of the TBtools software (Version 2.019). The locations of BEVs in the banana genome were determined by blasting. The genome sequence of the banana was then converted into chromosome length by TBtools software’s Fasta Stat (Version 2.019). Gene density profiles for the entire genome were generated based on gene structure annotation, resulting in a GFF3/GTF file. After that, the BEVs integration sites, chromosome length, and total genome density information were entered into Gene Location Visualize (Advanced) to visually represent the locations of the BEVs on the banana chromosomes.

### 4.6. Collinear Analyses of BEVs in Banana Genome

The collinear map of the BEVs on the AA, BB, and SS bananas was generated using the locations of the BEVs on the banana genome and the chromosome length of the banana genome by the extended Circos function of TBtools (Version 2.019).

## 5. Conclusions

Our results indicated that the distribution of BEVs among different genotypes of bananas was uneven and associated not only with banana genotypes but also with the copies present in the bananas. A higher number of BEVs were observed in A and B heterozygous bananas compared to those in A or B homozygous bananas. The phylogenetic analyses showed that the BSVs and BEVs were grouped into three clades; Clade II had the majority of BEVs, while a few found in Clade I. Furthermore, the distribution of BEVs across the AA, BB, and SS chromosomes of the bananas varied; BEVs tended to cluster together on the banana chromosomes to form hot spots. BEVs and BSVs shared five conserved motifs, and BEVs had two conserved amino acids that were different from BSVs. However, BEVs integrated into banana genomes at different times. Some of them integrated prior to the differentiation of the banana ancestor, while others after. Our findings suggested that BDF, consisting of BSVs, BEVs, and eBSVs, together drove the coevolution of bananas and BSVs. BDF is a potential threat to banana production, but it also serves as a powerful weapon for bananas against BSVs, and how to use this double-edged sword remains unknown.

## Figures and Tables

**Table 1:** Distribution of BEVs on the Chromosomes of *M. acuminata* subsp. DH-Pahang (AA).

BEVs	Loci	Chromosomes
GZ20	8	Chr03, Chr04, Chr08, Chr09
GZ23	38	Chr01, Chr02, Chr03, Chr04, Chr05, Chr07, Chr08Chr09, Chr10, Chr11
NGA	15	Chr01, Chr03, Chr05, Chr06, Chr09
UC	5	Chr05, Chr06, Chr10
UD	23	Chr01, Chr02, Chr04, Chr05, Chr07, Chr08, Chr10
UF	25	Chr01, Chr02, Chr03, Chr04, Chr06, Chr10, Chr11
UH	3	Chr01, Chr11
UG	2	Chr09

**Table 2:** Distribution of BEVs on the chromosomes of *M. balbisiana* subsp. PKW (BB).

BEVs	Loci	Chromosomes
GZ1	1	Chr05
GZ3	2	Chr09
GZ4	1	Chr11
GZ5	1	Chr05
GZ6	8	Chr07, Chr09
GZ7	2	Chr03, Chr07
GZ8	8	Chr02, Chr06, Chr09, Chr10
GZ11	6	Chr02, Chr03, Chr06
GZ15	2	Chr01, Chr10
P	14	Chr02, Chr03, Chr05, Chr06, Chr10
Q	16	Chr03, Chr04, Chr05, Chr06, Chr08, Chr10
UC	16	Chr02, Chr03, Chr05, Chr06, Chr07, Chr09
UF	1	Chr01
UG	28	Chr01, Chr02, Chr03, Chr05, Chr06, Chr07, Chr08, Chr09, Chr10, Chr11

**Table 3:** Banana samples in this study.

Banana Cultivars	Genotypes	No.	Collection Time	Collecting Place	Isolates
GY28	AA	1	December 2021	College of Horticulture, SCAU	GDGZGY28
Gongjiao	AA	3	March 2022	Institute of Fruit Tree Research, GDAAS	GDGZGJ1–3
Neiguijiao	AA	1	March 2022	Institute of Fruit Tree Research, GDAAS	GDGZMG1
Huangdijiao	AA	2	October 2022	Dongguan, Guangdong	GDDGHD1–2
Hongjiao	AAA	2	October 2022	Dongguan, Guangdong	GDDGHJ1–2
Guifeijiao	AAA	2	October 2022	Dongguan, Guangdong	GDDGGFE1–2
Nantianhuang	AAA	1	April 2021	Maoming, Guangdong	GDMMNTH1
Nantianhuang	AAA	1	April 2021	Kaiping, Guangdong	GDKPNTH1
Weiliansi	AAA	1	December 2021	Maoming, Guangdong	GDMMWL1
Baxijiao	AAA	1	December 2021	College of Horticulture, SCAU	GDGZBX1
Baxijiao	AAA	1	September 2021	Guangzhou, Guangdong	GDGZBX2
Baxijiao	AAA	1	April 2021	Dongguan, Guangdong	GDDGBX1
Baxijiao	AAA	1	April 2021	Kaiping, Guangdong	GDKPBX1
Baxijiao	AAA	1	February 2021	Yunnan Province	YNBX1
Qujiangyejiao	BB	1	March 2022	Institute of Fruit Tree Research, GDAAS	GDGZQJ1
Huashiyejiao	BB	1	March 2022	Institute of Fruit Tree Research, GDAAS	GDGZHS1
Jinshaxiang	AAB	1	March 2022	Institute of Fruit Tree Research, GDAAS	GDGZJSX1
C.La.Nang Tiem	AAB	2	March 2022	Institute of Fruit Tree Research, GDAAS	GDGZC1–2
Dajiao	ABB	1	December 2021	College of Horticulture, SCAU	GDGZDJ1
Fenjiao	ABB	1	December 2021	College of Horticulture, SCAU	GDGZFJ1
Jinfen	ABB	2	April 2021	Guangxi Academy of Agricultural Sciences	GXNJF1–2
Yuekangfen 1	ABB	2	September 2021	Guangzhou, Guangdong	GDGZYKF1–2
Guangfen 1	ABB	1	April 2021	Maoming, Guangdong	GDMMGF1
Guangfen 1	ABB	1	September 2021	Guangzhou, Guangdong	GDGZGF1
Fenza 1	ABBB	1	September 2021	Guangzhou, Guangdong	GDGZFZ1
Fenza 1	ABBB	8	April 2021	Guangzhou, Guangdong	GDPYFZ1–8
Fenza 1	ABBB	2	April 2021	Guangzhou, Guangdong	GDNSFZ1–2
Fenza 1	ABBB	2	September 2021	Fosan, Guangdong	GDFSFZ1–2
Malaijiao	Unknown	2	April 2021	Maoming, Guangdong	GDMMBM1–2
Yunjiao	Unknown	1	February 2021	Yunnan Province	YN2
Majiao	Unknown	2	October 2022	Dongguan, Guangdong	GDDGMJ1–2
Unknown	Unknown	6	February 2021	Greenhouse, SCAU	GDWS1–6
Unknown	Unknown	7	February 2021	Farm, SCAU	GDNC1–7
Unknown	Unknown	3	February 2021	Huizhou, Guangdong	GDHZ1–3
Unknown	Unknown	5	May 2021	Luoding, Guangdong	GDLD1–5
Unknown	Unknown	4	May 2021	Yunfu, Guangdong	GDYF1–4
Unknown	Unknown	2	May 2021	Zhangjiang, Guangdong	GDZJ1–2
Unknown	Unknown	4	May 2021	Maoming, Guangdong	GDMM1–4
Unknown	Unknown	2	May 2021	Beihai, Guangxi	GXBH1–2
Unknown	Unknown	3	May 2021	Beiliu, Guangxi	GXBL1–3
Unknown	Unknown	3	May 2021	Qinzhou, Guangxi	GXQZ1–3
Unknown	Unknown	3	May 2021	Wuzhou, Guangxi	GXWZ1–3
Unknown	Unknown	6	May 2021	Yulin, Guangxi	GXYL1–6
Unknown	Unknown	4	February 2021	Hainan Province	HN1–4

GDAAS: Guangdong Academy of Agricultural Sciences; SCAU: South China Agricultural University.

## Data Availability

The data presented in this study are available in the article.
